# THESEUS: The European research priorities for human exploration of space

**DOI:** 10.1038/npjmgrav.2016.34

**Published:** 2016-10-13

**Authors:** Joan Vernikos, Nicolas Walter, Jean Claude Worms, Stéphane Blanc

**Affiliations:** 1 Life Sciences, NASA HQ, Washington, DC, USA; 2 European Science Foundation, Strasbourg, France; 3 Université de Strasbourg, IPHC, Strasbourg, France; 4 CNRS, UMR7178, Strasbourg, France

Today, the exploration of space remains one of the most stimulating and exciting areas of scientific research and technological development. One of the objectives for the next 10 years is to create, and then implement, a long-term plan for the robotic and human exploration of the solar system, with Mars and the Moon as first targets. To undertake such a future mission requires major efforts of global and interdisciplinary cooperation between scientific, industrial, and legislative parties. This was recently highlighted in the report by the Committee on Human Spaceflight of the National Academy of Sciences of the United States of America.^1^

Past space missions in Earth orbit have demonstrated that humans can survive and work in space for durations of up to several months and return to Earth with relatively limited health consequences. However, there are pending technological, medical, and psychological issues to be solved before adventuring in longer and more distant space missions, such as those envisioned in a space exploration program. For example, protection against ionizing radiation, problems with lunar and Martian dust, a reliable closed life-support system in transit and on the surface, psychological issues such as those affecting cognition, behavior, and performance during and after long-duration space travel, general metabolic disturbances such as prevention of bone loss and muscle atrophy, and the potential irreversibility of these changes, as well as balance and coordination as the main limiting factors for a manned mission to Mars. Similarly, the known enhanced infectious disease risks as humans travel and live within these stressful, confined environments, require special attention. Furthermore, technological breakthroughs, primarily in life-support systems and recycling technologies are required to reduce the costs of these expeditions to more acceptable levels. Solving such issues will need creative scientific and technological approaches relevant to clinical and industrial applications here on Earth.

In the United States, NASA Human Research Program implements a constantly evolving Human Research Roadmap that represents a “risk reduction strategy for human space exploration.”^2,3^ In Europe, European Space Agency (ESA) supported several preliminary studies enabling the definition of priorities to prepare for interplanetary-manned exploration missions, such as the HUMEX (study on the Survivability and Adaptation of Humans to long-duration Exploratory Missions)^4^ or FIPES (Facility for Integrated Planetary Exploration Simulation).^5^ ESA has also developed a comprehensive and targeted ground program to validate effective countermeasures through the bed rest studies, including the testing of the use of artificial gravity applied through a short-arm centrifuge. However, Europe had not developed its own human exploration roadmap. On the basis of the current knowledge, an action plan approved by the European scientific and industrial communities, and relying on ESA programs, integrating the expertise of non-ESA member states as well as of the European Eastern countries is needed.

In 2012, the European Union funded the project THESEUS (Towards Human Exploration of Space: A European Perspective, supported by the EU Seventh Framework Program for Research and Technology Development). The goals of THESEUS were to develop an integrated life sciences research roadmap enabling European human space exploration, in synergy with the ESA strategy, taking advantage of the expertise available in Europe, and identifying the potential of non-space applications and development. As any human exploration initiative can only be conceived of as a well-coordinated series of programs at the international level, the issue of international cooperation and coordination with other nations, agencies, and programs has been central to the establishment of such a roadmap. Space agencies are currently jointly discussing their plans for exploring the Moon and Mars.^6^ Any research roadmap enabling human exploration of these targets should, both, incorporate these plans in its making, and influence such plans to ensure that basic and applied research priorities as defined and expressed by the relevant communities are addressed in a consistent manner by these agencies. To enable this, the concerned space agencies were represented as observers in THESEUS and international experts were invited to participate in the expert groups.

THESEUS had three objectives: (i) identify disciplinary research priorities, (ii) focus on fields with high-terrestrial application potential, and (iii) build a European network as the core of this strategy. These three objectives were tackled through five tightly interrelated clusters involving 137 international scientists:

- Integrated Systems Physiology
- Psychology and Human-Machine Systems
- Radiation
- Habitat Management
- Health Care

The conclusions of those expert groups can be found online on the THESEUS website.^7^ A series of publications is forthcoming in *NPG Microgravity* dedicated to the reports condensed as reviews of the cluster on Integrated Systems Physiology.

Why did we decide to publish this topic as a series of reviews? The actual human research program is the culmination of the 40-year space flight experience, including both short- and medium-term duration missions. The scientific results from these missions have demonstrated that space flights have an impact on almost all physiological systems including muscle atrophy, bone demineralization, cardiovascular and metabolic dysfunctions, impaired cognitive processes, and reduced immunological competence, on which undernutrition is superimposed. All these physiological responses lead to a physiological deconditioning in space that may affect crew health and performance, and thus the success of the mission, but also that may interfere with a healthy return to Earth. Countermeasures were investigated to mitigate some of the maladaptive changes associated with space flight. These included drugs, nutrition, and physical exercise on various workout devices and others. None of them were proven to be fully effective, but given the relative short duration of the missions, most astronauts returning to Earth did not encounter difficulties in recovering. Significant knowledge has undoubtedly been accumulated on the physiological changes associated with the adaptation of humans to short-term space flights.

Nowadays, human space flight programs have entered the next phase of space exploration toward the Moon and Mars, and there are clearly inherent medical challenges with such a goal. We have clearly much less information regarding the physiological changes associated with the long-duration missions extending from 30 days to months in orbit than we have for short-term missions. Preliminary observations from limited number of astronauts exposed to 6 months in space now suggest that our previous hypotheses that systems would have adapted to the space environment within this period, are not proving to be correct.

Clearly, the primary thrust of the next bioastronautics research will be to further explore the rate and magnitude of the effects of long-duration space flight on crew health and performance, to develop more effective and efficient countermeasures based on specific understanding of the mechanisms of sensing and responding to microgravity, and to facilitate post-flight readaptation to the terrestrial environment. Such basic and upstream research is a clear prerequisite activity aimed at improving, in the long term, the capability for interplanetary travel and life on planetary surfaces.

This objective is far from being trivial. Although space medicine has been practiced for more than half a century, it is quite nascent, relative to the physiological and clinical capability, and knowledge required for long-term space flight. For example, a significant challenge in the evolution of space medicine will be to determine how the physiological adaptation to space may alter the physiopathology of disease, or the manifestation of illness and injury in space. An answer to that can obviously be found by reviewing the clinical experience of flight physicians who treated diseases in space. But the strongest approach is to create an integrated model of the physiopathological adaptation of multiple organ systems to microgravity. The exploration of space requires a systematic understanding of human body, from the molecular to integrated system levels, as it responds to the unfamiliar environment of space travel.

This integrated physiological approach is definitely a new but necessary method in bioastronautics. Also, it has been recognized for a long time, though not executed as a required scientific line of attack. Research has for decades been conducted along body functions, i.e., muscle, bone, cardiovascular system, or nutrition taken independently. The best examples to demonstrate the limits of such an approach lie in the incomplete success of the current countermeasure programs, in which scientists focused on some organ systems and symptoms in a piecemeal manner rather than targeting at once in an integrated manner all of the body physiological systems affected by gravity. The association between the negative energy balance and both the decreased protein synthesis rate and the cardiovascular deconditioning, or the dual effect of amino acid supplementation that appears helpful on muscle mass but not on bone, demonstrate this point. A holistic approach to space-condition adaptation should now be developed.

The transition of space research from independent physiological functions to a more rigorous integrated approach requires a focused, competitive research strategy for solving targeted risk areas of human health and performance. Reaching these goals will not only provide the basis for critical, high-quality health care for crews on orbit, as well as a smooth recovery on return, but also result in a wealth of physiological data. Evaluation of these data will undoubtedly help addressing medical challenges of long-term space flight. The data will provide the basis for well-conceived and evidence-based decisions to such physiological concerns as radiation exposure, vestibular dysfunction, immunology, mineral metabolism, protein synthesis, chronobiology, and sleep, cardiology as well as food and nutrition in space, taken as a whole. Another essential consideration for planetary exploration is reduced gravity as opposed to weightlessness. The effects of a long-duration exposure to the reduced gravity levels that will be experienced during stays on the Moon (0.16 g) and Mars (0.38 g) are completely unknown. It seems unlikely that the countermeasures developed on board the International Space Station, will adequately protect crews journeying to Mars and back over a 30-month period, or prevent the effects of a long-duration exposure to reduced gravity on the Moon or Mars. There is therefore a need to get basic knowledge on the physiological adaptation to reduced gravity levels, which will, again, be used to design an “integrated countermeasure” for preventing the detrimental effects of weightlessness or reduced gravity on the physiological systems of the body. The advantage presented for the first time by the systematic study of microgravity in space, Moon, Mars, and Earth gravity as a continuum will for the first time enable better understanding of how the human body as well as other organisms sense and process gravity.

Finally, the full use of ground-based applications of bioastronautics promise to yield a wealth of knowledge. For decades, clinicians and physiologists working in space research have worked separately without awareness or taking advantage of potential strong mutual questioning. Good examples for that is research on aging and chronic diseases. In our search of the environmental factors that fueled the pandemic of chronic diseases, we face a paradox. Although sedentary lifestyle has been highlighted for decades as one of the main factors triggering the development of current chronic diseases such as obesity, insulin resistance, hypertension, muscle disuse, and bone demineralization, the physiology of physical inactivity has received little attention. Clearly, the causal relationships between sedentary behaviors and those disorders are essentially based on epidemiological studies or on the indirect beneficial effects of exercise training. None of these studies provide evidence to support a cause-and-effect relationship, but they indirectly suggest that sedentary behaviors and poor nutrition are the second leading cause of death in the United States, right after tobacco, and major contributors to the diseases associated with aging.^8^ Along with microgravity, the physiological adaptations to space flight require an adaptation to physical inactivity and lack of postural change that is exceedingly well simulated in the ground-based bed rest analog of microgravity.

In addition to space medicine-related questions, bed rest represents a unique model to investigate the mechanisms by which physical inactivity and lack of postural change leads to the development of current societal chronic disorders, but also to test the efficacy of the countermeasure programs in preventing or reducing the development of these disorders. More importantly, because bed rest studies are conducted in healthy subjects who are expected to recover, new therapeutic avenues can be studied.^9^

In the context of integrated systems physiology, THESEUS considered the following objectives:

- To delineate what are the next priorities of high-quality research in physiology needed to support the next phase of space exploration;
- To define these priorities in a new integrated approach of the deconditioning syndrome based on which a new generation of countermeasures may be tested in the context of Mars exploration;
- To better use the results of space physiology and their analogs to strengthen our knowledge of the role of sedentary behaviors on Earth in the development of current societal chronic diseases.

Five reviews are included. They are dedicated to the fields of nutrition and metabolism, immunology, muscle and bone, neurophysiology, and cardiovascular function. All of them made a significant effort to address each topic from an integrated physiology perspective. Finally, a concluding review summarizes the roadmap and compares it with the existing US-NASA roadmap.
